# COVID-19 in Switzerland real-time epidemiological analyses powered by EpiGraphHub

**DOI:** 10.1038/s41597-022-01813-5

**Published:** 2022-11-17

**Authors:** Flávio Codeço Coelho, Eduardo Corrêa Araújo, Olivia Keiser

**Affiliations:** 1 Fundação Getulio Vargas, Escola de Matemática Aplicada, Rio de Janeiro, 22250-900 Brazil; 2 The Global Research and Analysis for Public Health (GRAPH) Network, Geneva, Switzerland; 3 University of Geneva, Institute of Global Health, Geneva, Switzerland

**Keywords:** Respiratory tract diseases, Databases

## Abstract

Here we present the design and results of an analytical pipeline for COVID-19 data for Switzerland. It is applied to openly available data from the beginning of the epidemic in 2020 to the present day (august 2022). We analyzed the spatio-temporal patterns of the spread of SARS-CoV2 throughout the country, applying Bayesian inference to estimate population prevalence and hospitalization ratio. We also developed forecasting models to characterize the transmission dynamics for all the country’s cantons taking into account their spatial correlations in COVID incidence. The two-week forecasts of new daily hospitalizations showed good accuracy, as reported herein. These analyses’ raw data and live results are available on the open-source EpiGraphHub platform to support further studies.

## Introduction

The COVID-19 pandemic had a positive impact on the availability of daily disease surveillance data all over the world^1^. This made it possible to design analytical pipelines that could yield valuable insights into the day-to-day dynamics of the disease. One of the key analyses that this data has supported was the forecasting of diverse metrics related to the pandemic, such as new cases, hospitalizations etc. The recent literature contains a large number of publications on forecasting the course of the epidemic and extensive reviews have also been published^2,3^. Most previously published models are mechanistic transmission or statistical models. Not many describe or propose improved data analysis pipelines to automate the entire process from data collection to the generation of predictions.

Based on the available Swiss data, we have conceived and built a data collection and analysis pipeline, that produced daily updated analyses of the situation while keeping all the original data and results of the analysis on an open data platform for future reference and reproducibility^4^. In doing that we adhered to the FAIR data principles(www.go-fair.org): to make data *Findable*, *Accessible*, *Interoperable* and *Reusable*.

Here, we present a set of analyses defined by our research group that led to daily updated predictions of new cases and new hospitalizations, which were then shared with health professionals and other stakeholders dealing with the pandemic response. Besides the forecast models we also present intermediary transformations applied to the data to improve the models’ outputs. The analyses presented here can be applied to any other country with minor modifications if the required data are available. Initiatives like ours may help to stimulate access to open data that was fostered by the COVID pandemic^5^. Morgan *et al*. (2021) also indicate that many challenges remain to continue improving the access to disease surveillance data. We hope that our work can serve as evidence of what is possible when free flow of information is guaranteed between government and academia.

The main focus of this paper is to introduce a wide variety of analyses made possible by our data integration and analysis pipeline which is available as open source software. They range from Bayesian estimation of epidemiological indicators to spatio-temporal correlation and forecasting models.

## Methods

### Data sources

**Fig. 1:**
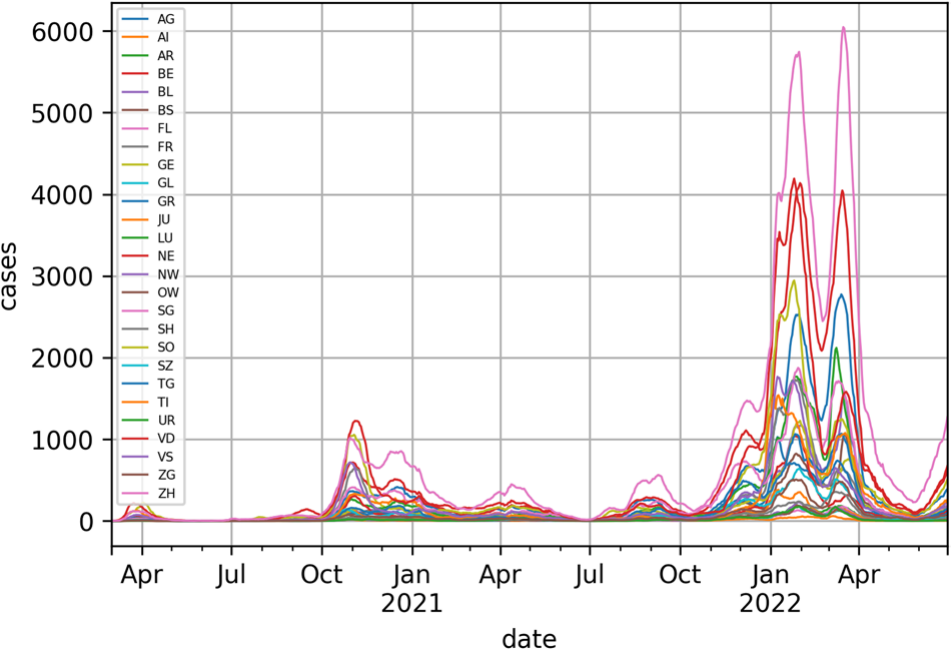
Daily case counts for all cantons. We can see that there are time delays between their incidence curves. Cantons are identified the their official two-letter codes (en.wikipedia.org/wiki/ISO_3166-2:CH).

For this work, we have used data from Switzerland’s Federal Office of Public Health (FOPH - opendata.swiss/en/dataset/covid-19-schweiz). We used reported numbers of daily cases (shown in Fig. 1), hospitalizations, tests, test positivity, and deaths from all 26 cantons. Cantons are administrative areas of Switzerland similar to a state in the United States.

The datasets used here, are listed in Table 1 where we describe the names, brief descriptions and links to where they can be accessed within the EpigraphHub platform^4^. For each of the datasets, a corresponding metadata table is also available (https://epigraphhub.org/tablemodelview/list/). Metadata tables have the same name as the tables they refer to, with the added suffix “*_meta*”. Other COVID-19 datasets obtained from the Swiss federal office of public health are also available for download and visualization in EpigraphHub. The datasets are stored and re-shared without modifications.

**Table 1:** List of datasets used for this paper.

Dataset name	Description	Link
foph_cases_d	Number of cases	https://epigraphhub.org/superset/explore/table/28/
foph_hosp_d	Number of Hospitalizations	https://epigraphhub.org/superset/explore/table/48/
foph_test_d	Number of tests performed	https://epigraphhub.org/superset/explore/table/93/
foph_hospcapacity_d	Hospital beds occupied	https://epigraphhub.org/superset/explore/table/56/
ngboost_forecast_hosp_d_results	Hospitalization forecasts	https://epigraphhub.org/superset/explore/table/113/

Additional datasets can be found on EpigraphHub under the Switzerland schema. Dataset names ending in *d* are updated daily.

For a summary visualization of the datasets used here, see epigraphhub.org/superset/dashboard/p/yorXv7eBJAQ/.

### Visualizing hospitalization rates

**Fig. 2:**
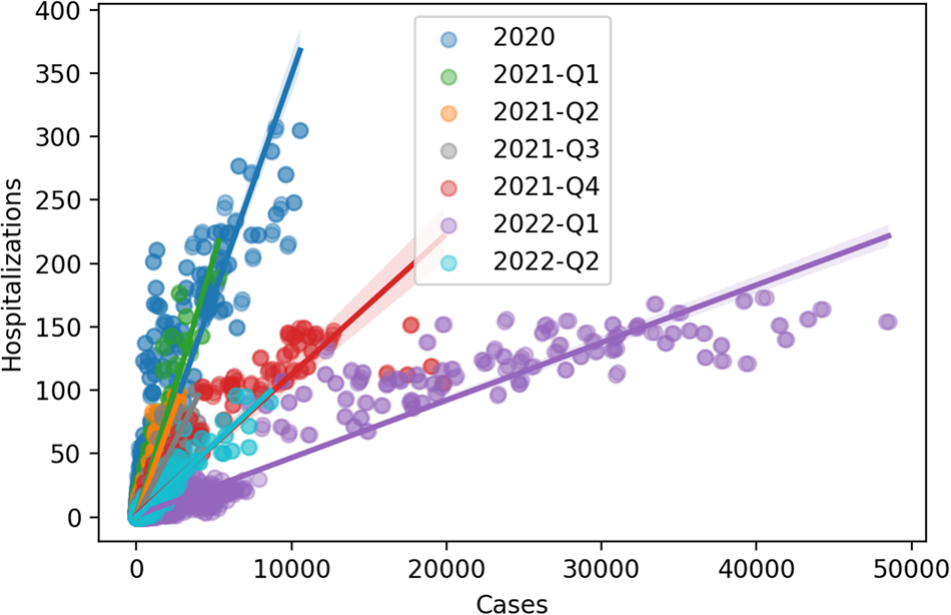
Daily Hospitalizations by cases in Switzerland, coloured by quarters (3-month windows). Q1: January-March, Q2: April-June, Q3: July-September, and Q4: October-December. Trend lines represent the average ratio of hospitalization per case.

**Fig. 3:**
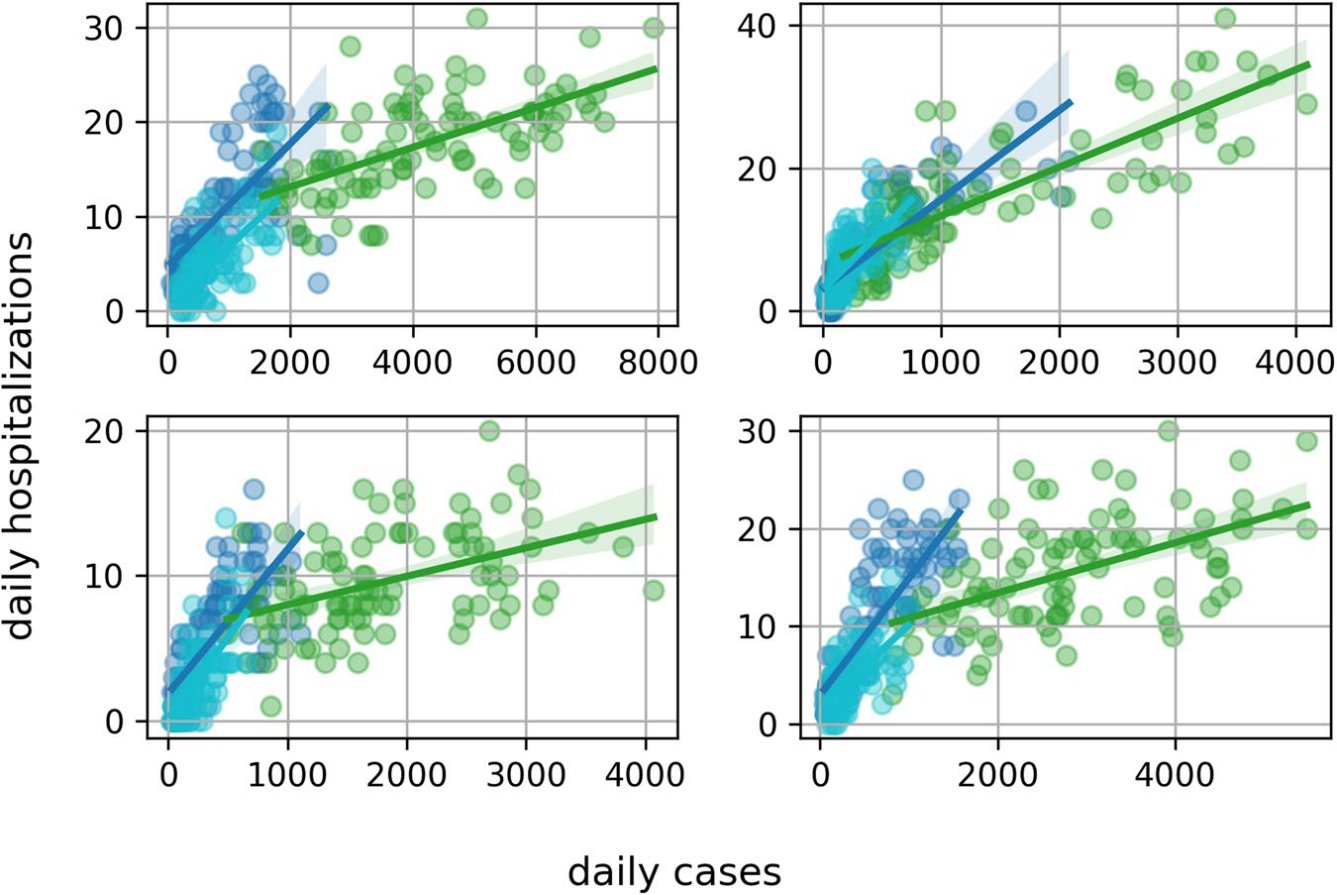
Daily Hospitalizations by cases in Zurich, Geneva, Aargau and Bern. Blue circles are from Q4 of 2021, green ones from Q1 of 2022 and cyan for Q2 of 2022.

Given the succession of viral variants and the effects of vaccination from the beginning of 2021, a simple yet effective way to visually follow the evolution of hospitalization risk over time is to look at the day-to-day relationship between the number of new hospitalizations and daily reported number of new Covid-19 cases. This visualization can be achieved by a simple scatterplot and applying a temporal colour mapping. Finally, we split the analysis into 3-months blocks to show the evolution of the average severity of cases (Fig. 2). We can look at these rates by canton, to see how they differ from the national rates (Fig. 3).

### Spatio-temporal analysis

From the daily reported case time series for each canton, we applied pair-wise correlation analysis to unravel the spatial dynamics of the virus.

As the virus spreads through geographical regions (e.g. cantons), the delays in the incidence of reported cases in different regions can be estimated (Fig. 1). We used cross-correlation between the daily reported new case series to estimate not only this spatial trajectory over time, but also analyzed the magnitude of the the pair-wise correlations between all cantons. To obtain the lag *τ* between two series, we assessed the lag that maximized the cross-correlation coefficient between every pair of cantons.

The normalized cross-correlation function for two time-series, *X*_*t*_ and *Y*_*t*_ is given by:

1 $${\rho }_{XY}(\tau )=\frac{{\mathbb{E}}\left[\left({X}_{t}-{\mu }_{X}\right)\left({Y}_{t+\tau }-{\mu }_{Y}\right)\right]}{{\sigma }_{X}{\sigma }_{Y}}.$$

The sign of *τ* that maximizes the cross-correlation function is a proxy of the direction of predictability, i.e., if *ρ*_*XY*_(*τ* > 0) it means that canton *X* anticipates *Y* in incidence trends, and can thus be a good predictor for *Y* ^6^. To find the value of *τ* that maximizes the correlation for each pair of cantons, we calculated *ρ*_*XY*_(*τ*) for values of *τ* ranging from −30 to 30 days. Here, *μ* and *σ* are the mean and standard deviation for each time-series. We used this information for building forecasting models for each canton, as shown below. Of note, this measure is no evidence of causation between pairs of geographical regions^7^. However, it allows to select the regions which can contribute to short-term predictions of trends in others regions.

### Spatial clustering

**Fig. 4:**
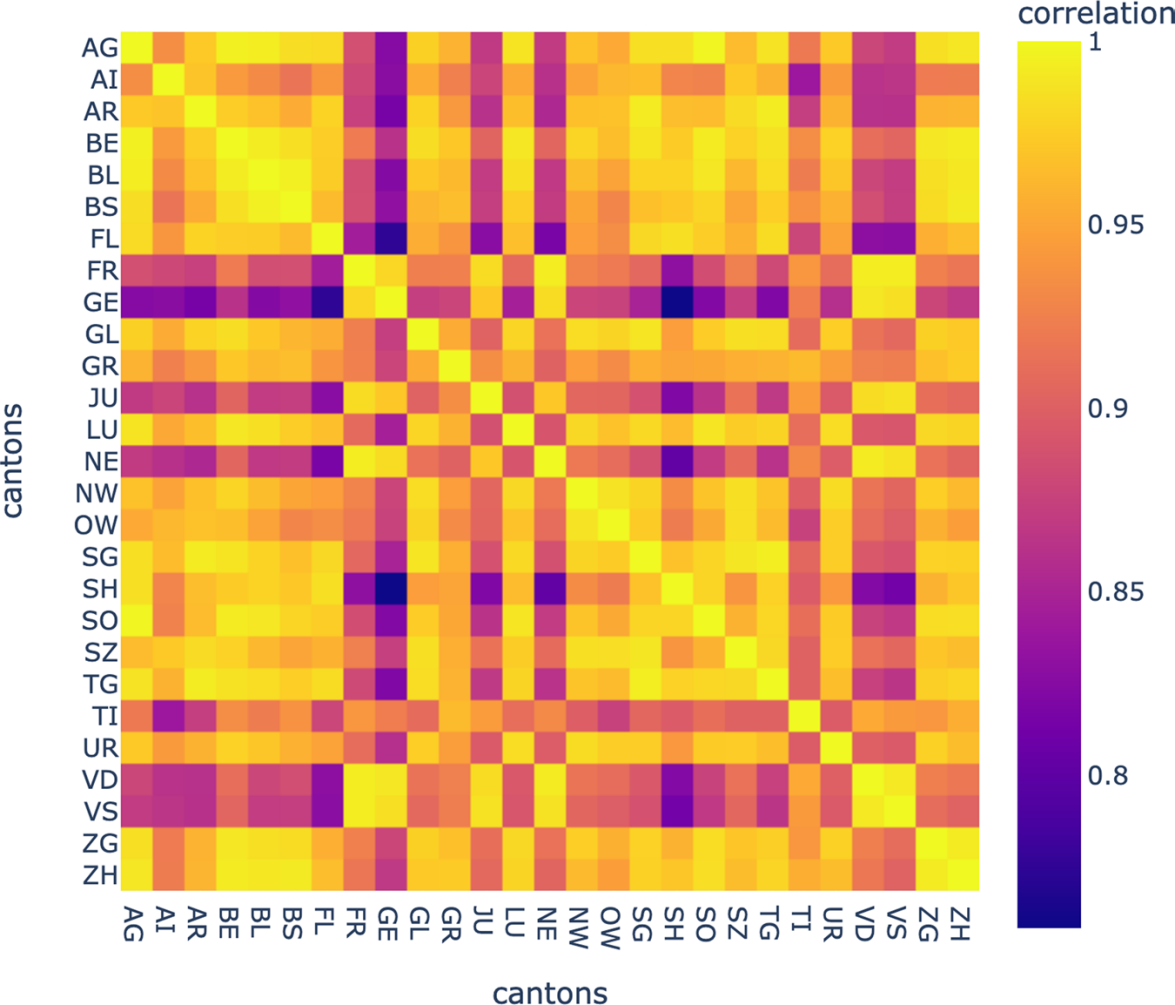
Cross-correlation matrix between cantons’ incidence time series.

**Fig. 5:**
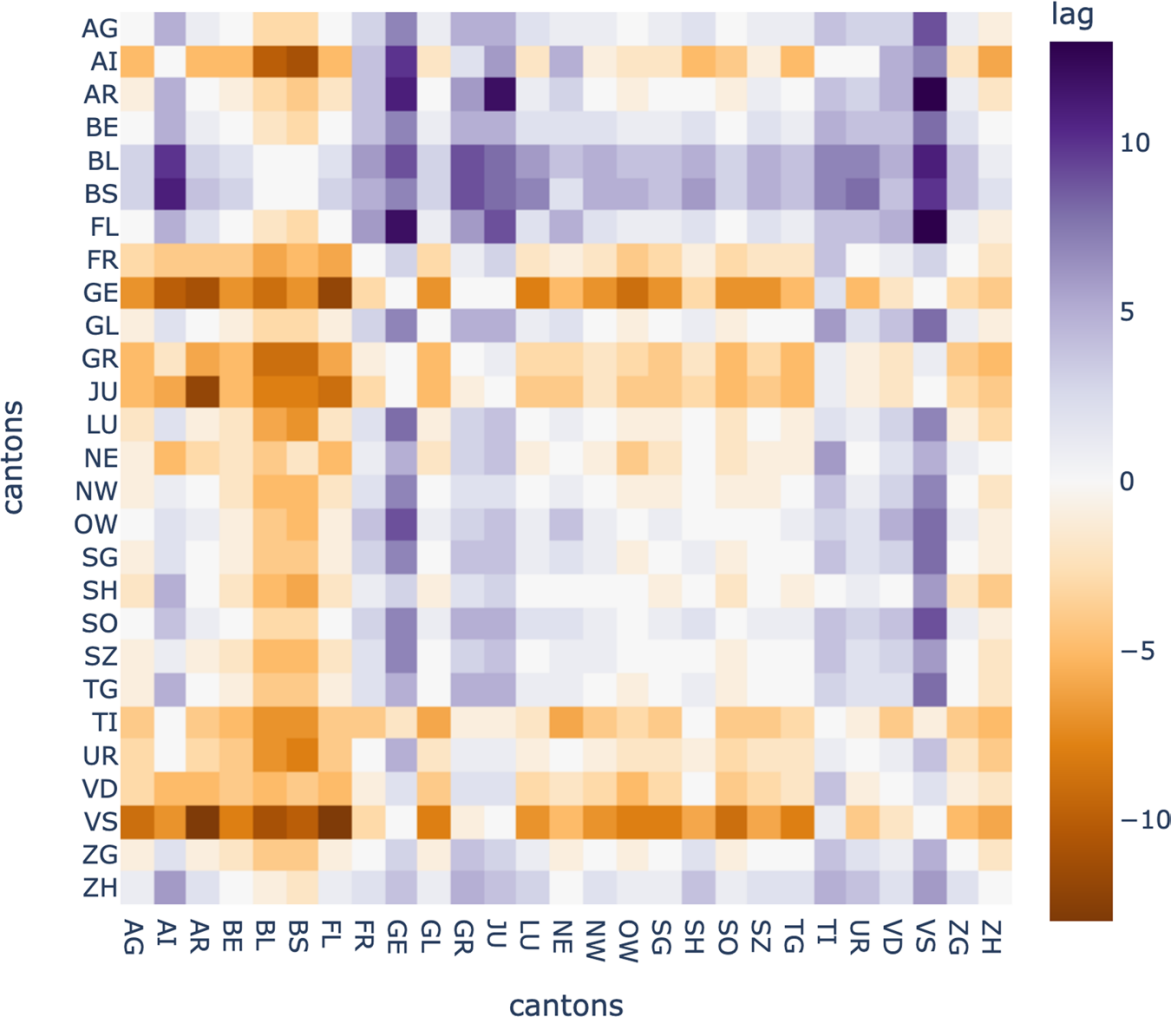
This matrix shows the lag that maximizes correlation between each pair of cantons.

By using the 1−*max*(*ρ*_*XY*_) as a distance between cantons *X* and *Y*, we can perform an agglomerative clustering of the cantons^6^, taking into consideration the optimal lag obtained as described above. Maximal correlations and optimal lags are stored as correlation and lag matrices, respectively (Figs. 4, 5).

### Estimation of prevalence and hospitalization rate

If we think about epidemics as stochastic processes, we can use the available data to make inferences about their rates. Here we used the cases, tests and hospitalization series to estimate prevalence and hospitalization rates.

Estimating prevalence from the reported number of cases is not trivial, as the testing frequency varies substantially over time, influencing the number of cases detected. Thus, we built a simple Bayesian hierarchical model to estimate prevalence of infection *Pv*_*t*_ and hospitalisation rate *Ph*_*t*_ from the number of tests *T*_*t*_ and the number of positive tests (*Cases*_*t*_).

We start by modeling the reported cases (*Cases*_*t*_) as a Binomial process (Eq. 2) with parameters *n* and *p* corresponding to the number of daily tests and the fraction of positive tests, respectively^8^.

Suppose we assume that the number of tests and the testing coverage are big enough so that the population tested approximates a representative sample of the general population. In that case, the number of positive tests will allow us to estimate the probability of a test being positive. This can be used to approximate the proportion of infected individuals in the general population, i.e. the prevalence, *Pv*_*t*_. To get a proper representation of the prevalence, we can model it using a Beta prior: $P{v}_{t} \sim Beta\left({\alpha }_{p},{\beta }_{p}\right)$, technically, treating it as a random variable.

2 $$Case{s}_{t} \sim Bin\left(n={T}_{t},p=P{v}_{t}\right).$$

In a similar fashion, we can model the probability of Hospitalization as $P{h}_{t} \sim Beta\left({\alpha }_{h},{\beta }_{h}\right)$ and the Hospitalizations as a Binomial,

3 $$Hospitalization{s}_{t} \sim Bin\left(n=Case{s}_{t},p=P{h}_{t}\right).$$

The complete Bayesian model then becomes:

$$\begin{array}{lll}Hospitalization{s}_{t}| P{h}_{t} &  \sim  & {\rm{Bin}}(n=Case{s}_{t},p=P{h}_{t}),\\ Case{s}_{t}| P{v}_{t} &  \sim  & {\rm{Bin}}(n={T}_{t},p=P{v}_{t}),\\ P{h}_{t} &  \sim  & {\rm{Beta}}({\alpha }_{h},{\beta }_{h}),\\ P{v}_{t} &  \sim  & {\rm{Beta}}({\alpha }_{p},{\beta }_{p}).\end{array}$$

The choice of non-informative Beta priors, ${\alpha }_{h}={\beta }_{h}={\alpha }_{p}={\beta }_{p}=0.5$, was taken to start the inference from a neutral *a priori* perspective.

These simple probabilistic models have a closed-form expression for the posterior distribution of the Binomial probability parameters, as they are based on conjugate distributions (Beta-Binomial). The inference based on the models described here was done with the PyMC python package(www.pymc.io) or using the closed formulas for the posterior Beta distributions.

The advantage of having a probabilistic representation of the incidence, is that we can plug it into the Binomial model of hospitalizations (Eq. 3).

### Forecasting models

The utilization of ensemble models to forecast epidemiological time-series has been successfully applied many times in recent years^6,9^.

To forecast the cantons’ hospitalization curves, we used a probabilistic gradient boosting machine model^10^, as they can capture complex non-linear relationships in multiple time series regression models.

The model was defined as

4 $$\begin{array}{lll}ln{H}_{k,t} & = & {\beta }_{0,k}+{\beta }_{1,k}{C}_{k,t-{\tau }_{i}}+{\beta }_{2,k}{H}_{k,t-{\tau }_{i}}\\  &  & {+\beta }_{3,k}{T}_{k,t-{\tau }_{i}}+{\beta }_{4,k}IC{U}_{k,t-{\tau }_{i}}+\varepsilon ,\end{array}$$

where *H*_*k, t*_ is modeled as a log-normal random variable, is the number of new hospitalizations in canton *k* on day *t*, *C* is the incidence, *T* is the number of tests performed, and *ICU* is the number of ICU patients. Each of these predictors enters the model 14 times, with a lag *τ* = 1…14 (we use the last 14 days of each series as predictors). In the same way, various cantons from the same cluster as *k* are also added to the model with the same lags.

The model of Eq. (4), can be trained to predict hospitalizations for any day ≥*t*. Here we used it to forecast the number of hospitalizations up to 14 days ahead (Fig. 9).

The Forecast models are run daily, right after the data is updated in the EpiGraphHub database. The forecasts are then also saved in EpiGraphHub. URLs for the up-to-date data sets used and the results tables are given in Table 1.

## Results

A web dashboard was developed in the Python language, using the Streamlit framework (streamlit.io) to display all the results of the analyses. The dashboard is a a standalone application that connects to the EpiGraphHub Database to fetch the data and the results of the analyses. The dashboard is hosted at the EpigraphHub platform (epigraphhub.org/covidch/). The full source code for the dashboard is also available (github.com/thegraphnetwork/COVID-CH-dashboard).

Results for the analyses described above are shown and discussed below for a few selected cantons, but results for all cantons can be seen in the dashboard and are available for download from EpiGraphHub.

### Visualizing hospitalization rates

Hospitalization rates decreased throughout 2021 and into quarter 1 (Q1) of 2022 and then showed a rebound in Q2 2022 (Fig. 2).

Similarly, in individual cantons, there was also an apparent increase in COVID-19 hospitalizations in Q2 of 2022.

### Spatio temporal analysis

Figure 4 shows the results of the correlation analysis performed on the incidence data for each pair of cantons.

Figure 5 shows the matrix with the lag of the strongest correlation between each pair of cantons.

**Fig. 6:**
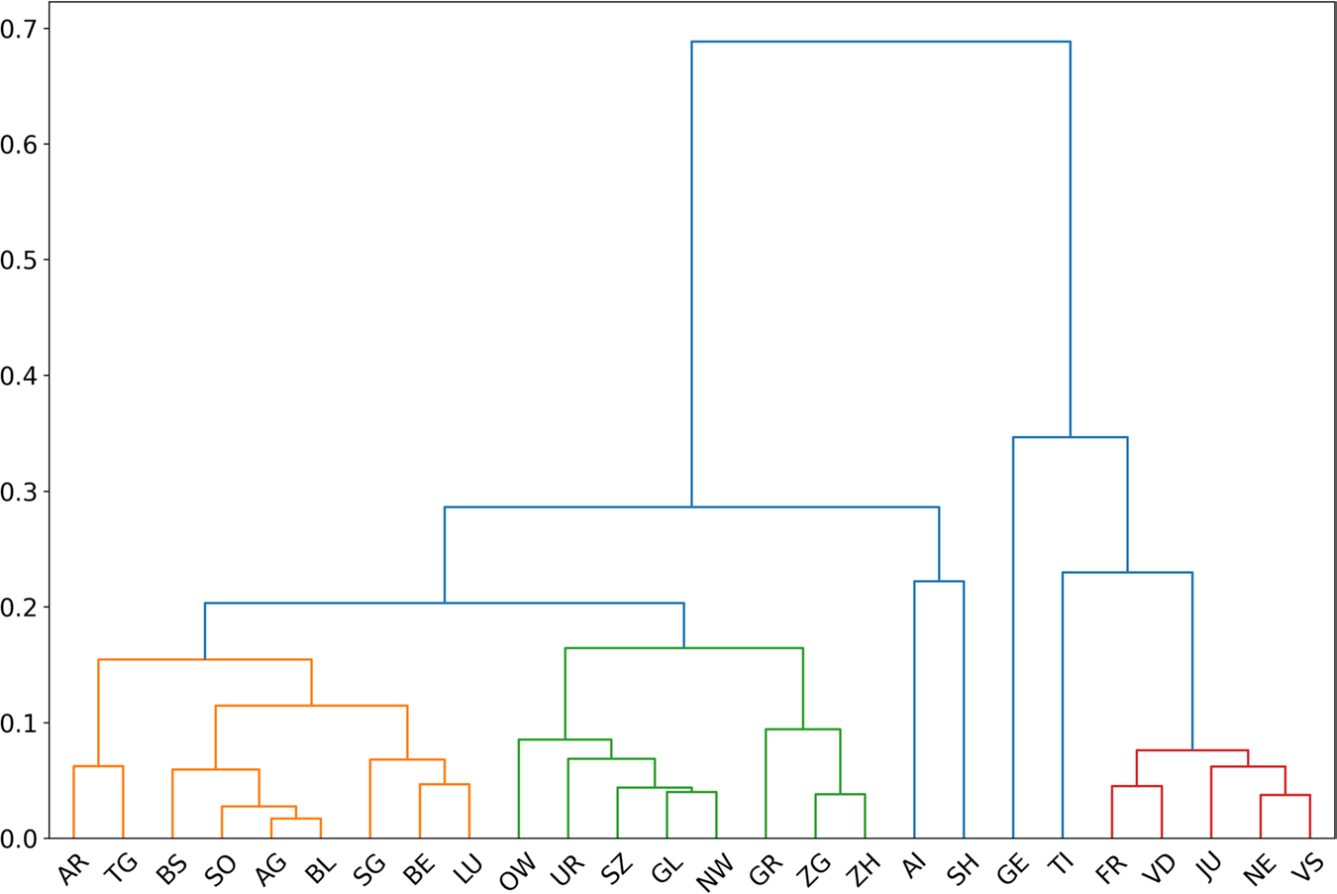
Result of the correlation clustering of the incidence time series.

Based on the correlation and lag matrices, we computed the clusters of cantons by correlation (Fig. 6).

### Estimation of prevalence and hospitalization rate

**Fig. 7:**
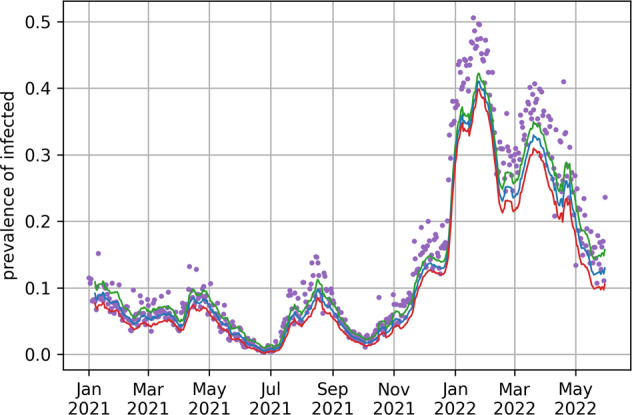
Estimated median prevalence of Covid-19 cases as a fraction of the exposed population in the canton of Geneva. Estimated median (blue line) and 95% credible bounds (red and green lines) are shown as a 7-day moving average. Purple dots are daily test positivity.

The posterior probability distribution for the prevalence over time in the canton of Geneva is shown in Fig. 7. Note that it matches the test positivity data (purple dots, not smoothed), which can also serve as a good proxy for prevalence.

**Fig. 8:**
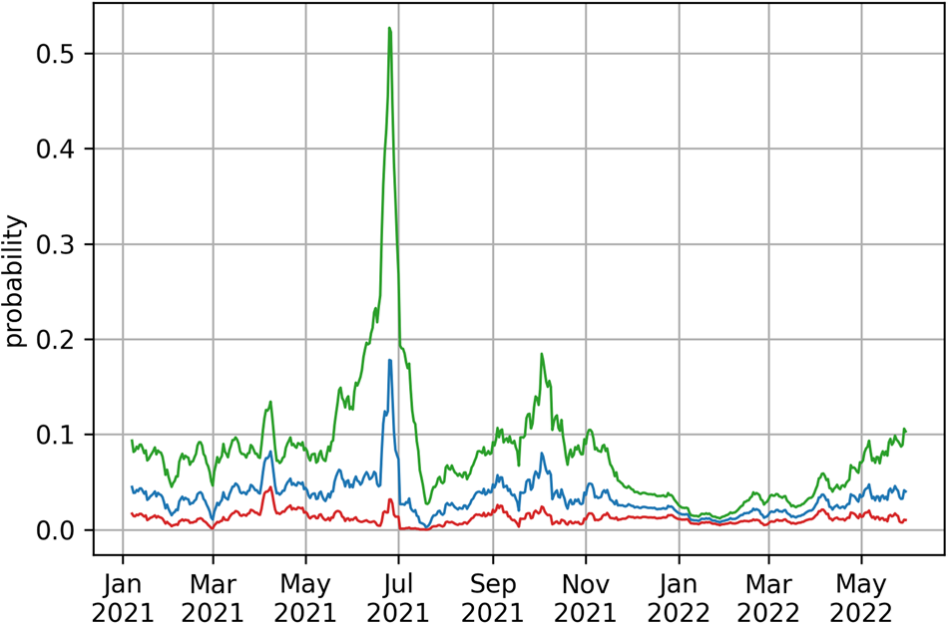
Posterior probability distribution for the probability of hospitalization in the canton of Geneva. Green and red lines represent the upper and lower bounds of the 95% credible interval, respectively. Blue line is the median.

The posterior distribution of the probability of hospitalization for positive cases for the canton of Geneva is shown in Fig. 8. The recent growth in hospitalization rates detected in Fig. 3, can also be seen here.

### Forecasting hospitalizations

**Fig. 9:**
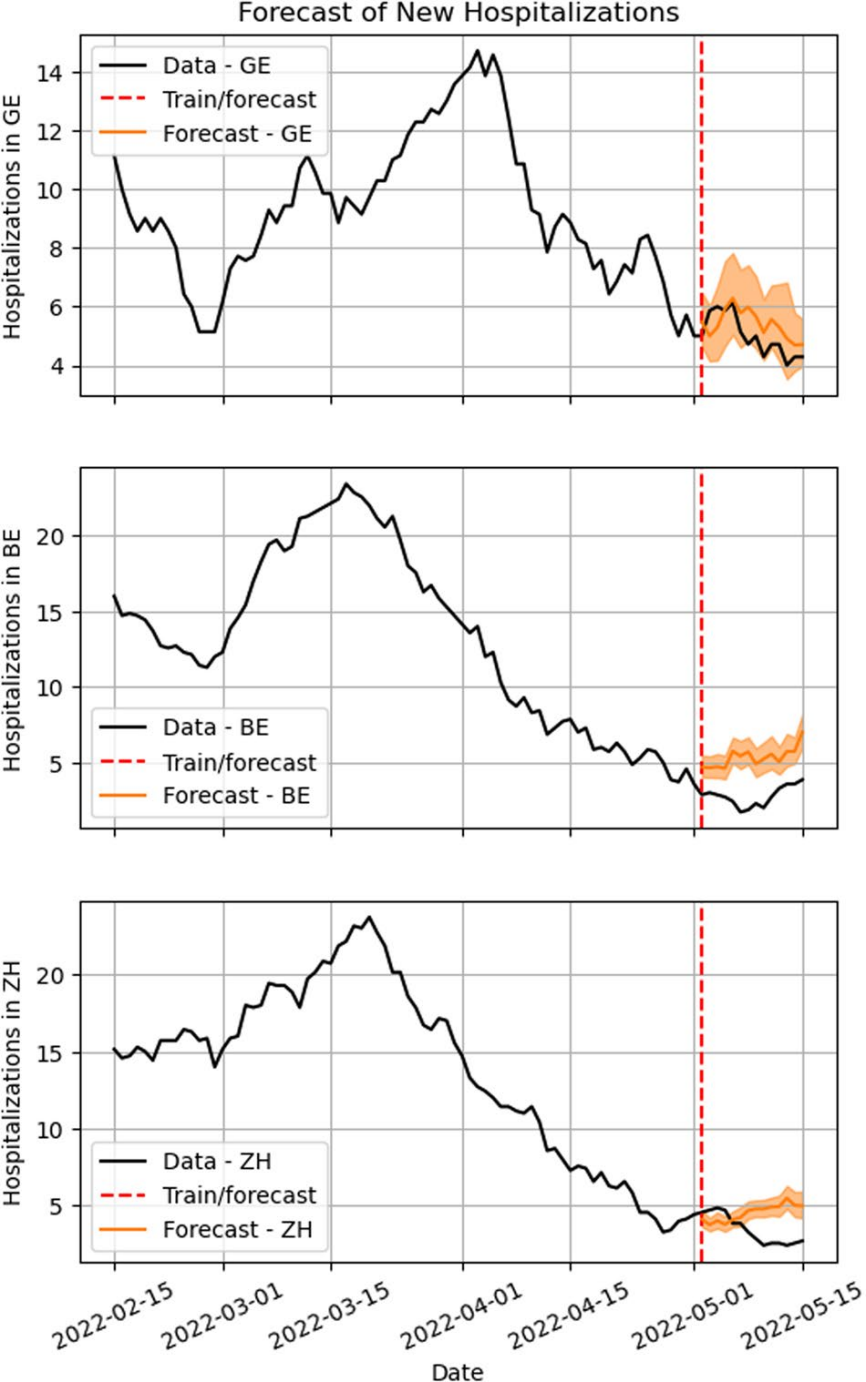
14-day forecast of new daily hospitalizations for the cantons of Bern and Zurich. Bern is shown on the left panel with a decreasing expectation of hospitalizations, and Zurich on the right with an expected increase.

The forecast of the number of new daily hospitalizations is shown in Fig. 9. The recent trend in the growth of hospitalization rates (Fig. 3) also appears in the 14-days forecast of the absolute number of daily hospitalizations in both Bern and Zurich. On Table 2, the *mean absolute percentage errors* both in sample (for the period used in model training) and out of sample (for data the model has not seen before), are reported for Bern, Geneva and Zurich.

**Table 2:** Mean Absolute Percentage Errors (MAPE) for the forecasts of cantons Bern, Geneva and Zurich, results are averages for the 14-day forecasts both in the training set (in sample), validation set (ou of sample) and actual forecasts.

Metric	Canton	In sample	Out of sample	Forecast
Mean Absolute Percentage Error	Bern	0.14	0.49	1.04
Mean Absolute Percentage Error	Geneva	0.17	0.39	0.13
Mean Absolute Percentage Error	Zürich	0.08	0.41	0.56

Note that MAPE are not actual percentages, but are rather the prediction error scaled as a fraction of the maximum value of *y* in the period, and thus can be greater than 1.

## Discussion

The availability of regularly updated and well-structured COVID-19 data from the Federal Office of Public Health of Switzerland was key to the realization of this work. Our previous work establishing the EpigraphHub platform was another critical component in enabling the quick deployment of the analytical pipeline and the online dashboard. The results assisted in planning the management of hospital beds when Covid-19 case numbers started to increase again, and there was a fear of another large wave of cases during the winter months.

During an infectious disease outbreak and a pandemic, quick access to well organized epidemiological analytical results is an important aid to decision-making. The analyses do not need to be complex, but provide details on overall trends, rates and spatial patterns that are not interpretable by using only raw data. The analytical pipeline described here was automatically executed within a few minutes after the data was made available daily, generating fresh forecast results. Recently however, the data update schedule has moved to weekly updates. The main advantage of having such an automated pipeline, is that no manual labour for generating a report by a professional is needed. The EpigraphHub platform was not only used to store the openly available data and results but also as a computational resource that allowed us to build and host the web dashboard quickly. The availability of such a platform should not be underestimated, since the work to fetch, transform, store and analyze the data every day is overwhelming to a single analyst on a personal computer. Besides having the code published as open-source software gives a level of transparency and reproducibility rarely offered by other initiatives^11^.

The Spatio-temporal correlation and clustering analysis revealed how to use data from other parts of the country to forecast the number of hospitalizations in a given canton. The selection of optimal predictors will likely change over time as causal factors governing transmission also change. This may cause the accuracy of forecasts to vary. So we recommend reassessing canton clustering regularly and considering including additional sources of information for maximal performance. Another possible improvement would be to look for the optimal geographical scale to describe the spread of the disease. The dataset we had access to, was aggregated by cantons which made this exploration impossible. One must also be aware that the most recent data used in such forecasting models are often underestimated due to delays in reporting^12^. The lack of individual level data with dates for the start of symptoms for each patient, makes it impossible to correct for testing/reporting delays.

The utilization of the daily testing data allowed for a more accurate estimation of prevalence. This estimation corrected for the variation in case detection rates, since the testing frequency depended on many non-epidemiological factors, such as travel and other time-varying testing recommendations. Additionally, the hospitalization rates are defined by the ratio between daily number of hospitalizations and new positive cases. When visualized by quarters (Fig. 2), this provided a clear view of the different phases of the pandemic in Switzerland highlighting the difference in severity between variants and also the effects of vaccination starting in 2021. An interesting variation of this approach would be to segment the series not by quarters, but by epidemiologically significant dates, such as start of vaccination, arrival of new virus variants of concern, etc.

The accuracy of the daily hospitalization forecasts was quite good (Table 2), especially when we consider that the model had no way to anticipate the arrival of new variants, from national data only. But the inclusion of information from the correlated cluster of cantons has be shown to help anticipating sudden changes in the incidence trends. This kind of model can continue to be improved if new data becomes available, particularly in the form of new predictore which could inform on strain evolution.

The relatively limited set of analyses presented in this paper highlights the significant potential for decision-making of having a tightly connected analytical pipeline to openly accessible data. The code for all the analyses is available from our GitHub repositories, as indicated in the code availability section. Making the data and results of these analyses also available through EpigraphHub not only contributes to transparency, but also encourages further analysis of the same datasets.

## Data Availability

All the data used in this paper is available through the EpigraphHub platform (epigraphhub.org/tablemodelview/list/?filters=(schema:(label:switzerland,value:switzerland))) and also on GitHub along with source code used in the analyses^13^.

## Code availability

The source code for all the analyses presented in this paper, and also the web dashboard, can be found on these GitHub repositories: github.com/thegraphnetwork/COVID-CH-dashboard and github.com/thegraphnetwork/epigraphhub_py. The first one for the web dashboard, and the second for the all the data collection and analyses. The documentation can be found on this link: epigraphhub-libraries.readthedocs.io.
